# Female fertility under the impact of COVID-19 pandemic: a narrative review

**DOI:** 10.1017/erm.2021.19

**Published:** 2021-11-02

**Authors:** Meng Wang, Bo Zhang, Lei Jin

**Affiliations:** Reproductive Medicine Center, Tongji Hospital, Tongji Medical College, Huazhong University of Science and Technology, Wuhan, China

**Keywords:** Assisted reproductive technology, COVID-19, female fertility, reproductive health cares, reproductive system, SARS-CoV-2

## Abstract

Coronavirus disease 2019 (COVID-19) is a serious respiratory disease mediated by severe acute respiratory syndrome coronavirus 2 (SARS-CoV-2) infection. The worldwide spread of COVID-19 has caused millions of confirmed cases and morbidity, and the crisis has greatly affected global economy and daily life and changed our attitudes towards life. The reproductive system, as a potential target, is at a high risk of SARS-CoV-2 infection, and females are more vulnerable to viral infection compared with males. Therefore, female fertility and associated reproductive health care in the COVID-19 era need more attention. This review summarises the mechanism of SARS-CoV-2 infection in the female reproductive system and discusses the impact of the COVID-19 crisis on female fertility. Studies have proven that COVID-19 might affect female fertility and interfere with assisted reproductive technology procedures. The side effects of vaccines against the virus on ovarian reserve and pregnancy have not yet been well investigated. In the future, the female fertility after SARS-CoV-2 infection and vaccination needs more attention because of the uncertainty of COVID-19.

## Introduction

Coronavirus disease 2019 (COVID-19) is a serious respiratory disease mediated by severe acute respiratory syndrome coronavirus 2 (SARS-CoV-2) infection (Ref. 1). COVID-19 has been identified as a pandemic by the World Health Organization. The worldwide spread of COVID-19 has caused millions of confirmed cases and morbidity, and the numbers are still increasing at an alarming rate (Ref. 2). SARS-CoV-2 is a pathogen with human-to-human airborne and aerosol transmission (Ref. 3), and the respiratory system, such as lung, is the main target for viral infection (Ref. 4). However, studies have also reported symptoms of other organs and systems, including the kidney, heart and reproductive system (Refs 5–7). Moreover, females are more vulnerable to viral infection compared with males (Ref. 8), putting females – in particular, females of childbearing age – at an increased risk of reproductive system impairment. Therefore, female fertility and associated reproductive health care in the COVID-19 era need more attention.

In this study, we summarise the mechanism of SARS-CoV-2 infection in the female reproductive system, review the impacts of the COVID-19 crisis on female fertility and discuss the current status of reproductive health care during the pandemic.

## Mechanism of infection in the female reproductive system

### Relationship between SARS-CoV-2 and ACE2

**Fig. 1.**
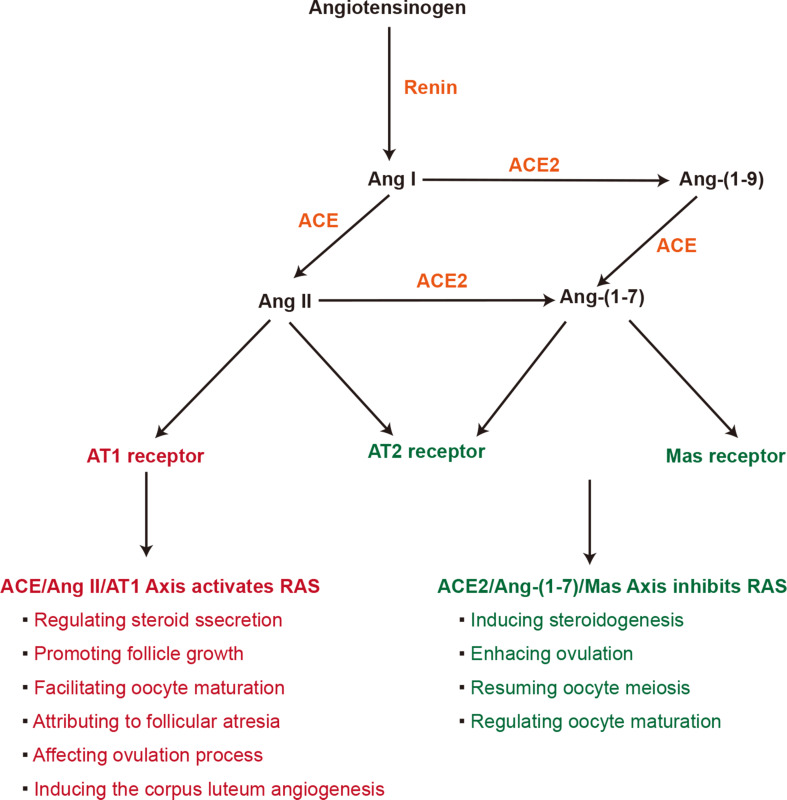
Components of RAS and its role in female ovarian function. Ang I, angiotensin I; Ang II, angiotensin II; ACE, angiotensin-converting enzyme; Ang-(1-9), angiotensin-(1–9); Ang-(1-7), angiotensin-(1–7); AT1, angiotensin II type 1; AT2, angiotensin II type 2; RAS, renin-angiotensin system.

Angiotensin-converting enzyme (ACE) 2, a homologue of ACE, is a zinc metalloprotease with hydrolase activity (Ref. 9) that is able to hydrolyse angiotensin (Ang) I and Ang II to generate Ang-(1–9) and Ang-(1–7), respectively (Ref. 10). Ang II and Ang-(1–7) hormones are the most important hormones produced in the renin-angiotensin system (RAS) and have opposite effects (Ref. 11). Ang II induces vasoconstriction and inflammatory reactions (Ref. 12), promotes proliferation (Refs 13, 14) and facilitates fibrosis and tissue remodelling (Ref. 15), whereas Ang-(1–7) has anti-inflammatory properties (Ref. 16), mediates vasodilation (Ref. 17) and alleviates cardiac and metabolic dysfunction (Refs 18–21). Thus, ACE2, a key component of the RAS, is essential to balance Ang II and Ang-(1–7) levels (Fig. 1).

The SARS-CoV-2 virus gains access to host cells via attachment to the ACE2 receptor (Ref. 22). Coronaviruses are spherical-enveloped viruses capsuled with positive single-stranded RNA. The structural proteins of SARS-CoV-2 are composed of spike (S), membrane (M), envelope (E) and nucleocapsid (N) proteins. The first three proteins are embedded in the viral envelope, whereas the N protein, a core component of the nucleocapsid, interacts with the viral RNA (Ref. 23). Similar to SARS-CoV-1, the viral S protein of SARS-CoV-2 has a strong affinity for ACE2 (Ref. 24). Viral S proteins have two subunits, the S1 and S2 domains. The S1 domain directly binds to receptors of host cells, whereas the S2 domain mediates viral and host cell membrane fusion (Refs 25, 26). This process is also facilitated by the proteolytic cleavage and activation of viral S proteins induced by the transmembrane protease serine 2 (TMPRSS2) in the cytoplasm (Ref. 27). Then, viral genomic RNA is released into the target cell cytoplasm and replicates using the host cell organelles, resulting in new virion release (Refs 28, 29). SARS-CoV-2 infection has been proven to decrease the activity and downregulate the expression of ACE2, resulting in an increase of Ang II recruitment and a decrease in Ang-(1–7) production in circulation, which explains the inflammatory reactions investigated in COVID-19 patients (Refs 30, 31).

### ACE2 and ovarian function

It has been reported that ACE2 exists in a variety of mammalian ovaries, including rats (Ref. 32) and cattle (Ref. 33). Additionally, ACE2 be detected in ovaries of women of reproductive age (Ref. 34). ACE2 is highly expressed in stromal cells, theca cells and granulosa cells, as well as oocytes (Refs 32, 35). In the female reproductive system, ACE2 is predominantly enriched in the ovary (Refs 36, 37), making it a potential target organ for SARS-CoV-2 infection. Moreover, previous studies have demonstrated that ACE2 has been detected in embryos before the 8-cell stage and in trophectoderm cells of late blastocysts, and TMPRSS2 exists in embryos in the late blastocyst stage (Ref. 38), revealing a high SARS-CoV-2 infection susceptibility in peri-implantation embryos (Ref. 39).

Ang II, most abundantly expressed in granulosa cells (Ref. 40), regulates steroid secretion (Ref. 41), promotes follicle growth (Ref. 42), facilitates oocyte maturation (Ref. 43), contributes to follicular atresia (Ref. 44), affects the ovulation process (Ref. 45) and induces corpus luteum angiogenesis (Ref. 46). Although Ang-(1–7), found predominantly in theca-interstitial cells, induces steroidogenesis, especially oestradiol and progesterone production (Ref. 47), enhances ovulation (Ref. 48), resumes oocyte meiosis (Ref. 49) and regulates oocyte maturation (Ref. 50). In addition, ACE2 can be found in follicles in various developmental stages, and the expression levels are regulated by the secretion of gonadotrophin, revealing the possible relationship between ACE2 expression and female fertility. Moreover, the level of Ang-(1–7) in human follicular fluid has been proven to be positively related to the oocyte maturation rate. This evidence supports the significance of Ang-(1–7) levels in the oocyte maturation process (Ref. 50). Furthermore, the decrease in ACE2 activity induced by SARS-CoV-2 infection can increase circulating Ang II, which might alter ovarian function, influence the biological process of oocyte development and maturation, impact oocyte quality and ultimately impair fertility function (Ref. 36). In addition, Ang II recruitment also increases oxidative stress (Ref. 51), which may lead to inflammatory reactions and affect ovarian physiology and reproductive ability (Fig. 1).

### ACE2 and endometrial activity

The uterus – in particular, the endometrium – is essential for female fertility, and the components of the RAS can be found in the uterus, especially in endometrial epithelial and stromal cells (Refs 52, 53). This makes the endometrium more susceptible to viral damage (Ref. 54), which might induce embryo implantation impairment. Some studies have suggested that RAS component expression varies with the menstrual cycles (Refs 37, 52). ACE2 expression has been proven to be more abundant in the secretory phase than in the proliferation phase, and lower in stromal cells than in epithelial cells (Refs 55, 56). Moreover, the expression of ACE2 is reported to increase with female age (Refs 54, 57). This evidence indicates that older females in the secretory phase are likely to be more susceptible to endometrial infection compared with younger women in the proliferation phase.

Evidence has demonstrated that the maintenance of Ang II and Ang-(1–7) balance is critical for regulating menstrual cycles because of the significant role of RAS in angiogenesis and tissue remodelling. Ang II, with tissue remodelling properties, induces spiral artery vasoconstriction, facilitates endometrial regeneration, enhances stromal proliferation and initiates menstruation (Refs 58–60). SARS-CoV-2 infection in the uterus might disturb the Ang II/Ang-(1–7) balance, decrease Ang II expression levels and alter Ang II distributions in the uterus, which may cause severe endometrial and myometrial disorders (Refs 52, 61), such as dysfunctional uterine bleeding (Ref. 62). Moreover, several studies have reported an association between ACE2 expression and the prognosis of endometrial cancer (Refs 63, 64), revealing the significant role of ACE2 and RAS in uterine function.

### ACE2 and pregnancy

The placenta provides nutrient and oxygen exchange between the mother and foetus. Although limited studies have investigated and analysed RAS function in the placenta, all RAS components are expressed in the placenta (Ref. 65), even in human placental cell lines (Ref. 66). The RAS has been assumed to regulate placental function by several studies (Ref. 67). Additionally, ACE2 is ubiquitous in the human placenta (Ref. 68), the expression of which is even higher than that in the lung, indicating that the placenta might be a potential target for the viral infection. Interestingly, ACE2 levels differ in various areas of the placenta (Ref. 68). In placental villi, ACE2 expression levels are most abundantly detected in the syncytiotrophoblast, cytotrophoblast and vascular smooth muscle of primary and secondary villi (Ref. 69), whereas in the maternal stroma, ACE2 is found predominantly in invading trophoblasts, intravascular trophoblasts and decidual cells (Ref. 68). ACE2 can be detected from 6 weeks of gestation until birth, but it is also expressed differently throughout foetal development (Ref. 70). It has been proven that ACE2 levels increase in early gestation but decrease dramatically in late gestation (Refs 71, 72). Furthermore, the most highly expressed areas transfer from the decidual zone, luminal epithelium and glandular epithelium to the labyrinth placenta, amniotic epithelium and yolk sac epithelium during gestation (Refs 69, 73).

The RAS is mainly involved in balancing vasoconstriction and vasodilation and regulating foetal development during pregnancy, and RAS components are also reported to influence several other biological processes. Ang II facilitates trophoblast invasion and angiogenesis (Ref. 74), and the overexpression of Ang II may result in gestational hypertension, preeclampsia and eclampsia (Ref. 37). Excessive vasoconstriction in preeclamptic women induced by high Ang II levels is attributed to the reduction of blood and nutrition supply in foetuses (Refs 75, 76). Similarly, decreased serum Ang-(1–7) and increased plasma Ang II levels can be observed in women diagnosed with preeclampsia (Ref. 77). Moreover, decreased ACE2 and Ang-(1–7) levels in the placenta may induce intrauterine growth restriction (Ref. 73). Additionally, ACE2 knockout in mice during pregnancy can result in placental function disorders, such as placental hypoxia, and finally foetal growth retardation (Refs 78, 79). Furthermore, an aberrant Ang II/Ang-(1–7) ratio is associated with premature birth (Ref. 80) and cardiovascular disorders in adult offspring (Ref. 81), which could be attenuated by upregulating ACE2 in rats (Ref. 82).

## Impacts of COVID-19 on the female reproductive system

### COVID-19 and female fertility

Ovaries may be a potential target for SARS-CoV-2 infection (Ref. 36), although until now, the impact of viral infections on female fertility has been debated. Ovarian reserve is used to evaluate female fertility, and basal antral follicle count, anti-Müllerian hormone (AMH) and sex hormones, such as follicle-stimulating hormone, luteinising hormone, oestradiol, progesterone and testosterone, are the most frequently utilised indicators of ovarian reserve (Ref. 83). In addition, a regular menstrual cycle can also reflect ovarian reserve in women of reproductive age (Ref. 84). Li *et al*. analysed the clinical data from 237 females with a history of SARS-CoV-2 infection, and they found that nearly a quarter of the participants had menstrual cycle changes, including volume and duration changes, despite similar serum AMH and sex hormone concentrations in the compared cohorts (Ref. 85). Another study reported a negative association between serum levels of both AMH and oestradiol and the severity of viral infection (Ref. 86). However, no significant differences have been observed in women with non-severe and severe COVID-19 in terms of status, volumes or phases of menstrual cycles. Of note, COVID-19 may act as a potential risk factor for ovarian function and cause ovarian injury, including decreased ovarian reserve and hormone disturbance, in infected women (Ref. 87).

According to previous human oocyte transcriptome and proteome databases, ACE2 and TMPRSS2, the essential molecules for SARS-CoV-2 entry into host cells, are expressed in human oocytes from the in vitro fertilisation process (Ref. 88). Immunohistochemistry analyses in human oocytes, as well as pre- and peri-implantation embryos, have also reinforced the strong expression of ACE2 in human oocytes and blastocysts (Ref. 89). Nevertheless, no studies have systematically evaluated and reviewed the impacts of SARS-CoV-2 infection on human oocyte development potential to date. However, in light of the susceptibility of SARS-CoV-2 infection to early embryonic development, great attention should be paid to embryonic development potential in infected women. Whether COVID-19 might cause oocyte and embryo impairments remains elusive and needs further evaluation.

### COVID-19 and pregnancy

SARS-CoV-2 infection, which constitutes a threat to both the mother and foetus, may be associated with various pregnancy and neonatal complications (Refs 90, 91). Reduced ACE2 levels in gravidas after infection induce a rise in placental Ang II levels, which promotes vasoconstriction in the placenta, accompanied by an increasing risk of gestational hypertension, and ultimately preterm birth and intrauterine growth restriction (Ref. 80). A recent systematic review also concluded that gravidas with COVID-19 have a higher risk of maternal death and preterm birth, and their babies are more likely to be hospitalised in the neonatal department (Ref. 92). Currently, no evidence has clearly proven that COVID-19 causes placental dysfunction, whereas to avoid possible obstetric risks, more obstetricians and gravidas reportedly prefer caesarean section (Refs 91, 93). Additionally, because of the high expression of ACE2 in the kidney, COVID-19-associated acute kidney injury is quite frequent (Ref. 94), and renal failure subsequently serves as a risk factor for death in hospitalised patents, particularly critically ill patients (Refs 95, 96). A previous study has reported viral infection in renal tubular cells (Ref. 97) and increased ACE2 levels in the kidneys of pregnant mice (Ref. 98). Thus, maternal kidney function during pregnancy in infected women is worthy of our attention.

It has been reported that foetuses born to mothers diagnosed with COVID-19 tested positive for nucleic acid identification through nasopharyngeal or anal swabs a few days after birth (Ref. 99). Moreover, newborns of infected women exhibited elevated serum SARS-CoV-2 immunoglobulin (Ig) M levels 2 h after birth, indicating the probable occurrence of intrauterine infection (Refs 100, 101). These cases suggest that infants may be infected during gestation. Nevertheless, a systematic review of 936 neonates with maternal infection has found that only 27 of them (2.9%) had a positive viral RNA test, revealing that vertical transmission of SARS-CoV-2 has a low incidence (Ref. 102).

According to GeneCards, ACE2 exists in the female breast, providing an entry site for SARS-CoV-2 infection (Ref. 37). A study performed SARS-CoV-2 nuclei acid identification tests in breast milk specimens from three infected females, and one of them tested positive, revealing the possibility of maternal–infant transmission by breastfeeding (Refs 37, 103). Moreover, the immune system of neonates has not been fully established (Ref. 104), and close contact during breastfeeding may lead to a higher risk of potential viral infection. Two cases of neonatal infection caused by unprotected breastfeeding by new mothers diagnosed with COVID-19 have also been reported (Ref. 37). Thus, although breastfeeding can effectively reduce the risks of neonatal infections in the respiratory and gastrointestinal systems and metabolic disorders (Ref. 105), we still strongly recommend artificial feeding to infected mothers. If the mothers insist on breastfeeding, adequate disinfection of hands and mask wearing should also be encouraged before and during breastfeeding to minimise the chance of viral transmission through close contact (Ref. 106). In addition, a disinfected breast pump is also recommended.

### COVID-19 and human gametes and embryos

Limited studies have provided direct evidence of the impact of SARS-CoV-2 infection on human gametes and embryos until now. Wang *et al*. found that SARS-CoV-2 infection in females might not negatively affect female fertility and embryo development by analysing assisted reproductive technology (ART) data (Ref. 107). The study compared the embryo outcomes of females with and without a history of SARS-CoV-2 infection via propensity score matching and found that the ovarian reserves and ovarian responses between groups were similar, as were the proportions of mature oocytes, fertilised oocytes, high-quality embryos and available blastocysts. No significant differences were found in terms of clinical pregnancy rate or miscarriage rate. Although theoretically, human oocytes and embryos are at a high risk of viral damage, much about the crisis, including the impact on fertility, remains unidentified, and evidence of the direct impact of SARS-CoV-2 infection on gametes and embryos is lacking.

## Future of COVID-19: vaccine and female reproductive health

During the post-pandemic era, vaccinations against COVID-19 seem to be general and essential, and the potential impact of vaccines on human fertility and offspring health deserves our concern and attention. A study collected and analysed data from online search queries in Google regarding the COVID-19 vaccine and fertility after the announcement of the COVID-19 vaccine emergency use authorisation by the Food and Drug Administration of the USA. Interestingly, they found a dramatic increase ranging from more than 200% to nearly 3000% in fertility-related search volume, demonstrating an increasing concern about the side effects of vaccines on human fertility among the general public (Ref. 108). According to the vaccine platform, the existing vaccines against COVID-19 are mainly categorised into three types: mRNA vaccines, replication-defective live viral vectors and recombinant subunit-adjuvanted protein vaccines (Ref. 109). BNT162b2, a Pfizer/BioNTech mRNA SARS-CoV-2 vaccine with an efficacy of 95%, contains mRNA coding viral S proteins of SARS-CoV-2 that enter cells to mediate immune responses by antibody production (Ref. 110). An animal study has investigated the effects of BNT162b2 on female fertility and offspring development in rats. Female fertility evaluation, embryonic development and neonatal development were similar, and no adverse effects of BNT162b2 were detected between the control group and the vaccine group (Ref. 111). Similarly, a recent clinical self-controlled study included 36 couples undergoing ART treatments before and after BNT162b2 administration and compared patients' performance and ovarian reserve in Israel, and no differences were observed in terms of ovarian response, stimulation processes or embryological parameters (Ref. 112). Moreover, another study showed a similar follicular quality in BNT162b2-vaccinated and -unvaccinated women (Ref. 113). Furthermore, there is a lack of data on other types of COVID-19 vaccines on fertility, despite the fertility safety of BNT162b2 investigated by the current studies. Future studies with larger sample sizes and longer follow-up periods are required to validate the existing results.

Considering the potential placental damage caused by SARS-CoV-2 infection, vaccine safety in pregnancy is a question of debate. Notably, none of the current vaccine clinical trials were conducted on pregnant women. The Centers for Disease Control and Prevention released the data of a large survey on the safety of the BNT162b2 vaccine in March 2021 (Ref. 113). Among 55 million individuals who received the vaccination in the United States, approximately 30 000 became pregnant by February 2021. A total of 1815 gravidas receiving BNT162b2 vaccines were enrolled in the vaccine safety survey. No increased risk of obstetric complications, such as miscarriages and preterm birth, was reported in these enrolled participants. Moreover, no pregnancy-related adverse effects were reported in the majority of these gravidas. Thus, in the updated report, the American College of Obstetricians and Gynecologists recommended the COVID-19 vaccine to gravidas (Ref. 113). Recently, a randomised controlled trial was registered to investigate and evaluate vaccine safety in gravidas, and more trials on pregnant women should be carried out (Ref. 114).

In addition, the concern about whether neonates can benefit from the vaccination of mothers through placental antibody transfer is increasing. Neonatal Fc receptor (FcRn) mediates the circulating IgG transport from mothers to offspring across the placenta, and placental IgG transfer exhibits an upward trend throughout gestation (Refs 115, 116). Moreover, increased levels of FcRn and FCGR3 in the placenta induce selective transfer of antibodies, especially IgG1 antibodies, the most promising subclass of IgG antibodies in immunotherapy (Refs 117, 118). A recent study reported that SARS-CoV-2 infection induces an increase in competitive IgG and FCGR3A levels in the placenta, greatly compromising placental antibody transfer, compared with influenza and pertussis, especially in the third trimester (Ref. 119). Furthermore, the modification patterns of transferred SARS-CoV-2 antibodies differ from other diseases, exhibiting lower levels of antibodies with galactosylated modification in neonates (Ref. 119), which has certain guiding significance for the optimisation of placental antibody transfer. Considering a greater impairment of placental antibody transfer in the third trimester, the second trimester is recommended for vaccination against SARS-CoV-2. However, the effective and safe dosage and timing of vaccination during pregnancy need more evaluation.

## Conclusion

The COVID-19 crisis has greatly affected daily life and changed our attitudes towards life. It is likely to persist for years, and we have to bear it and learn how to coexist with the pandemic. The reproductive system, as a potential target, is at a high risk of SARS-CoV-2 infection. The subsequent effects on female fertility and reproductive health care cannot be ignored and warrant further investigation. In this review, female reproduction issues related to the pandemic have been addressed, including ovarian function, pregnancy and assisted reproductive care, and several studies have provided evidence that COVID-19 might affect female fertility and interfere with ART procedures. Moreover, the side effects of vaccines against the virus on ovarian reserve and pregnancy have not yet been investigated, and studies with larger sample sizes should be conducted to ensure the safety of these vaccines. In the future, the female fertility after SARS-CoV-2 infection and vaccination needs more attention because of the uncertainty of COVID-19.
